# Compositional Correlation between the Nanoparticle and the Growing Au-Assisted In_*x*_Ga_1–*x*_As Nanowire

**DOI:** 10.1021/acs.jpclett.1c02121

**Published:** 2021-08-04

**Authors:** Robin Sjökvist, Daniel Jacobsson, Marcus Tornberg, Reine Wallenberg, Egor D. Leshchenko, Jonas Johansson, Kimberly A. Dick

**Affiliations:** † Centre for Analysis and Synthesis, Lund University, Box 124, 22100 Lund, Sweden; ‡ NanoLund, Lund University, Box 118, 22100 Lund, Sweden; § National Centre for High Resolution Electron Microscopy, Lund University, Box 124, 22100 Lund, Sweden; ∥ Solid State Physics, Lund University, Box 118, 22100 Lund, Sweden

## Abstract

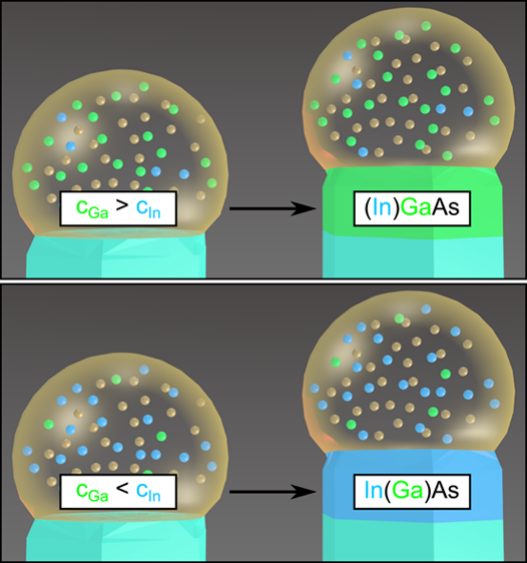

The nanowire geometry is favorable for the growth of ternary semiconductor materials, because the composition and properties can be tuned freely without substrate lattice matching. To achieve precise control of the composition in ternary semiconductor nanowires, a deeper understanding of the growth is required. One unknown aspect of seeded nanowire growth is how the composition of the catalyst nanoparticle affects the resulting composition of the growing nanowire. We report the first *in situ* measurements of the nanoparticle and In_*x*_Ga_1–*x*_As nanowire compositional relationship using an environmental transmission electron microscopy setup. The compositions were measured and correlated during growth, via X-ray energy dispersive spectroscopy. Contrary to predictions from thermodynamic models, the experimental results do not show a miscibility gap. Therefore, we construct a kinetic model that better predicts the compositional trends by suppressing the miscibility gap. The findings imply that compositional control of In_*x*_Ga_1–*x*_As nanowires is possible across the entire compositional range.

The creation of ternary III–V semiconductor nanowires is an important step in making nanowires suitable for advanced applications.^1^ While the properties of binary nanowires are fixed, the incorporation of a suitable third element from either group III or group V results in a material for which the properties are a tunable combination of those of the two participating binaries, depending on the ratio between them.^2^ This is due to III–V semiconductors being stoichiometric compounds, where each group V element forms a pair with a group III element in the solid, giving the formula A_*x*_B_1–*x*_V for a ternary compound with two group III elements, A and B. Nanowires are particularly promising compared to thin film growth, because lateral relaxation eliminates the need for a lattice-matched substrate and greatly increases the potential to selectively vary the composition. The variable composition increases the ability to tailor the nanowires to fit applications in the many fields where they can be used, including optoelectronics,^3−6^ quantum physics,^7,8^ and life science.^9,10^ InGaAs, which is a combination of InAs and GaAs, has, for example, been theoretically predicted to show high carrier mobilities with a small direct bandgap. This allows optical applications in the near- to mid-infrared region if the In concentration can be set to a constant, homogeneous value throughout the nanowire.^3,4,11^ Compositional control has, however, proven to be difficult, and only certain homogeneous compositions have been achieved for InGaAs.^12^

One of the two most common ways to grow epitaxial III–V semiconductor nanowires is through metal organic vapor phase epitaxy (MOVPE) [the other being molecular beam epitaxy (MBE)]. In this method, growth species are supplied as a vapor, and a liquid, metallic, nanoparticle is used to catalyze nanowire growth.^13^ The metallic nanoparticle commonly consists of Au. For growth to occur, one or more of the atomic growth species will have to dissolve in the Au nanoparticle, which will then nucleate the solid semiconductor at the growth interface. The ratio between the dissolved elements within the nanoparticle is expected to affect the composition of the growing ternary semiconductor nanowire, but the details are up for debate.

There have been several theoretical investigations concerning the compositional relationship between the liquid nanoparticle and the ternary nanowire. Among the theoretical models, there are two main approaches for describing the growth, depending on which process is considered to be the rate-limiting step. Kinetically limited growth^14,15^ is that in which the growth will be mainly governed by kinetic factors like incorporation rates, and nucleation limited growth^16−18^ is that in which the growth behavior will be determined by thermodynamics, e.g., the nucleation barrier. The obtained compositional relationships from the two approaches often have different appearances, meaning that the results can be contradictory. Notably, thermodynamic models often predict a miscibility gap, limiting the range of attainable nanowire compositions.

The nanoparticle and nanowire behaviors are difficult to study experimentally. Growth of nanowires in MOVPE has classically been a “black box” process, which means that most analysis is done after the growth has been performed. While the nanowire segments are expected to retain their composition after growth termination, the composition of the nanoparticle is expected to have changed. This is because the nanoparticle freezes from liquid to solid during growth termination, and the composition will change depending on the cooling process.^19^ Even if the composition of the nanoparticle would be retained, it gives only the end point, and the history of the nanoparticle would be lost, making it impossible to know what the nanoparticle composition was when different parts of the nanowire were grown. A solution to this problem is of course to measure the compositions of the nanowire and nanoparticle during growth, correlating them in time.^20^

In this paper, the growth of InGaAs nanowires has been studied *in situ* using an environmental transmission electron microscope (TEM). The nanowires were grown within the microscope from Au nanoparticles using an MOVPE system connected to the microscope, in which micro electro-mechanical system (MEMS) chips were used as the substrate and heat source. High-temperature X-ray energy dispersive spectroscopy (XEDS) was used, during growth, to measure the composition of the nanowires and their nanoparticles to determine their correlation. Finally, a kinetic model is proposed; it better predicts the experimentally measured correlation between the nanoparticle and the growing nanowire than previous models. The model shows that compositions throughout the entire compositional range are achievable, and that the arsenic concentration in the nanoparticle during growth has a significant effect on the compositional relationship between the nanoparticle and nanowire.

Figure 1 shows the systematic approach used for the experimental investigation of the nanoparticle and nanowire compositions. After growth initiation (see Experimental Methods), XEDS measurements of the nanoparticle, and the nanowire segment just beneath, were conducted separately. The procedure was to condense the electron beam to a small spot focused on the tip of the nanoparticle facing away from the nanowire (see Figure 1a), to record the spectrum of the nanoparticle. After the spectrum had been recorded (Figure 1c), the wire was allowed to grow for 5–10 min. By monitoring the growth rate (which was on the order of 0.1 nm/s, corresponding to 3.5 s per monolayer), we could determine the length of the freshly grown nanowire segment (Δ*L* in Figure 1b), where an XEDS measurement of the nanowire should be conducted. This was to ensure that the XEDS measurement of the nanoparticle was correlated with the correct part of the grown nanowire. Another point to note is that further down the nanowire in panels a and b of Figure 1, a widening due to shell growth (not catalyzed by the nanoparticle) is seen. To measure the composition of the nanowire that could be truly correlated to the nanoparticle, it was therefore important to condense the beam as close to the interface as possible, before the onset of shell growth. The spectrum related to the nanowire segment is shown in Figure 1d. The recorded spectra (Figure 1c,d) were then analyzed to extract and relate the concentrations of In and Ga in the nanoparticle to the nanowire, giving a time resolution on the order of a few minutes. The As and Au signals were carefully monitored for the nanoparticle and the nanowire, respectively, to make sure that the beam was condensed at a point sufficiently far from the interface as to not record signal from the other region. Arsenic has very low solubility in the Au droplet,^20^ and very little Au is incorporated into the nanowire;^21^ therefore, these signals clearly show whether the beam should be condensed further from the interface. A few measurements were discarded due to the As in the nanoparticle or the Au in the nanowire being measured as higher than 5 atomic percent, which was set as the cutoff limit. Details about a technique used to limit the scattered signal are given in the Supporting Information (SI 4: Distorted beam for XEDS). The measurement procedure was repeated several times for each nanowire and nanoparticle pair studied.

**Figure 1:**
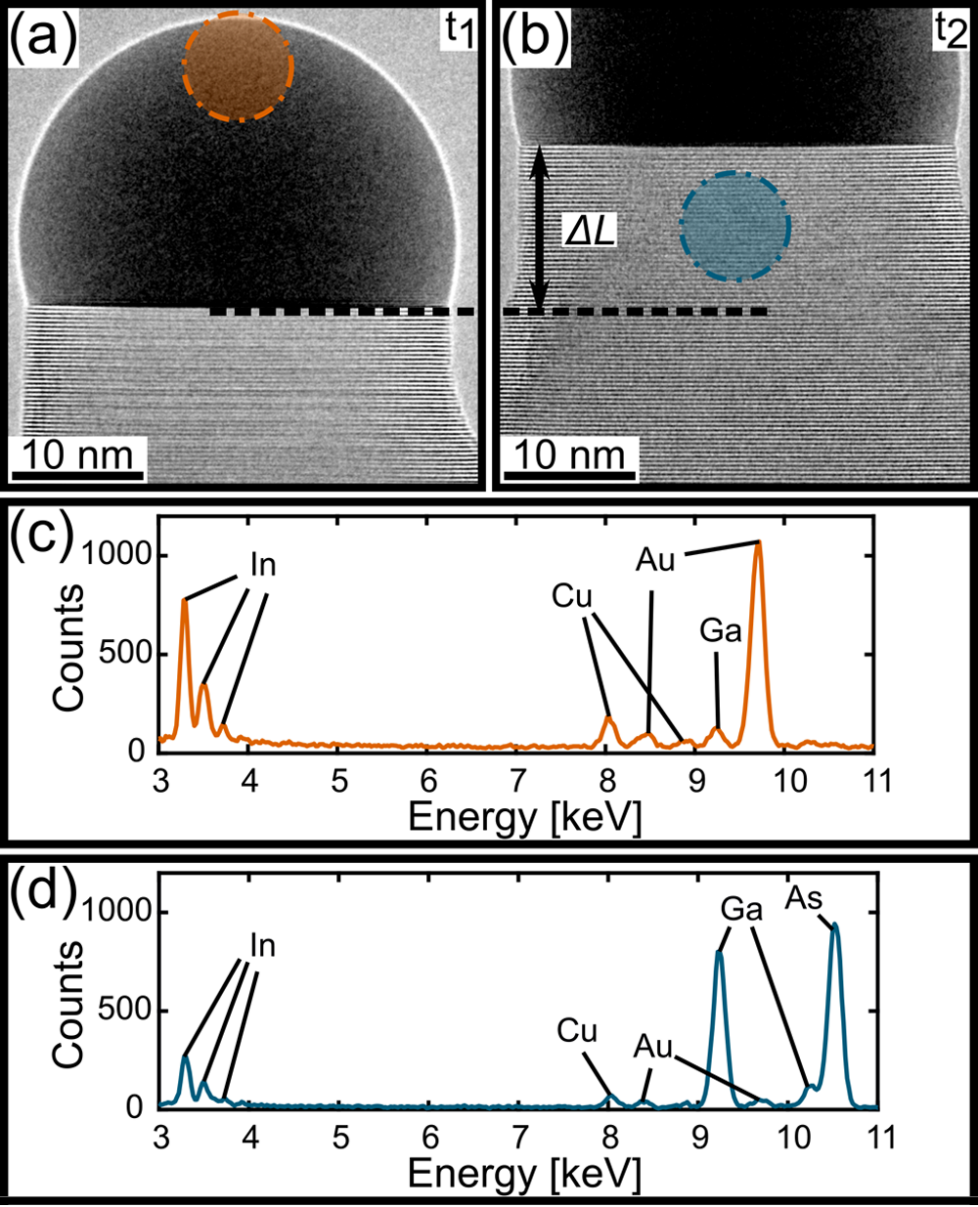
Procedure for the *in situ* XEDS measurements. (a) Growing nanowire at time *t*_1_ and a circular indication of where the beam was focused to measure the composition of the nanoparticle. The corresponding spectrum can be seen in panel c. (b) The same nanowire at some later time, *t*_2_, when the nanowire part has grown a distance Δ*L*, moving the nanoparticle upward in the figure. Upon examination of the distance the nanowire has grown, the area where the nanowire spectrum should be measured is located. The beam is focused in a limited area, shown by the circular region, and a spectrum is recorded and displayed in panel d. This procedure ensures that the spectra shown in panels c and d can be correlated in time.

In Figure 2a, the correlated compositions acquired from nanowires and nanoparticles are shown as red dots. On the *x*-axis, the measured nanowire composition is shown, represented as the In to total group III (In/III) ratio, and on the *y*-axis, the same ratio measured in the nanoparticle is presented. The total group III is, in this case, the summed concentration of In and Ga measured in either the nanowire or the nanoparticle. Each data point therefore represents a time-correlated measurement of a nanoparticle and its nanowire segment, and the graph contains measurements from many different nanowires grown in several separate experiments. Details about the raw compositional data treatment and interpretation are found in the Supporting Information (SI 2: The experimental data), and an error estimation is presented in SI 3: XEDS error estimation.

**Figure 2:**
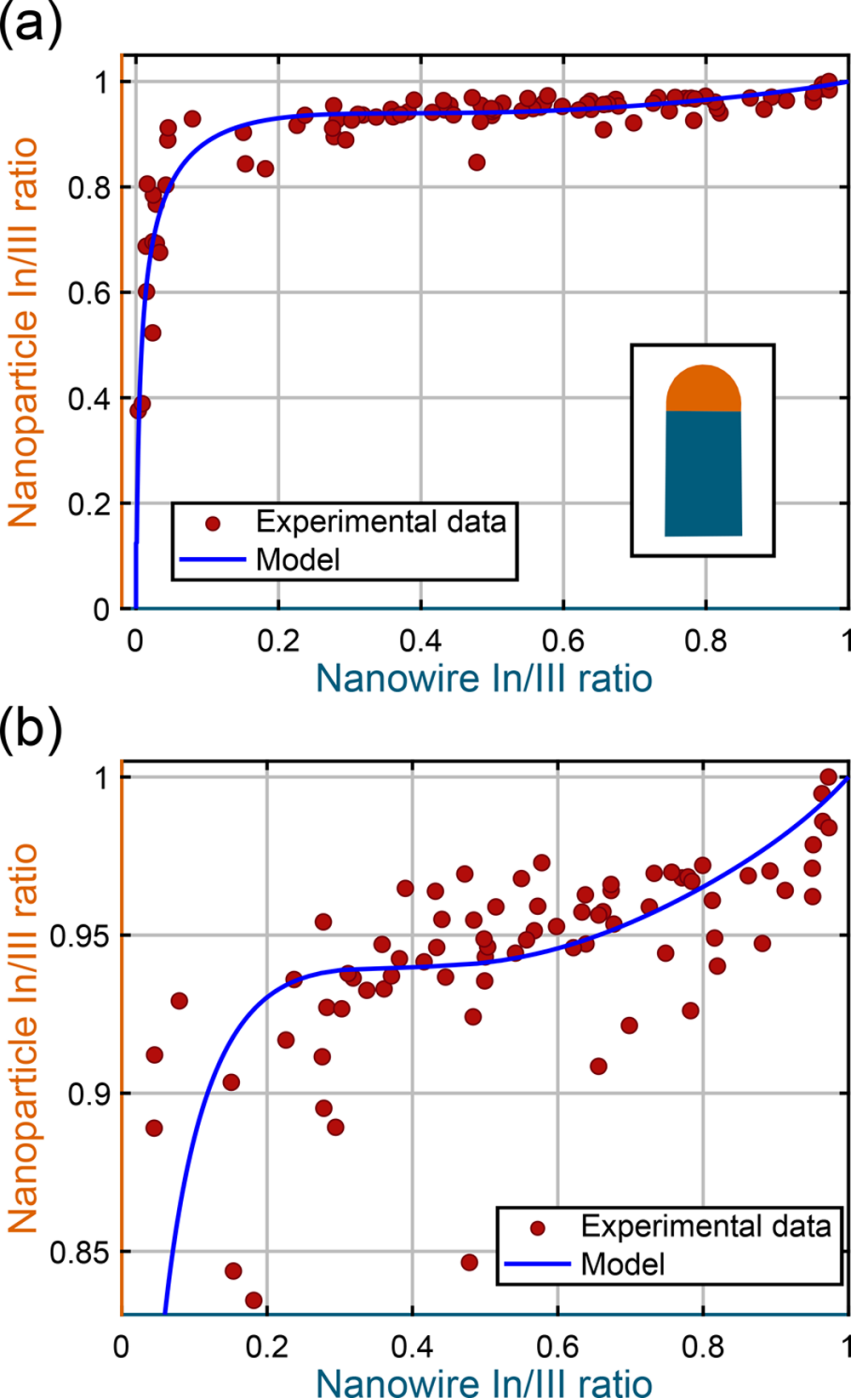
Time-correlated relationship between the nanoparticle and nanowire compositions, represented as In/III ratios. In panel a, the experimental data are presented as red dots and represent a compilation from the 15–20 analyzed nanowires. The theoretical model is plotted in blue, and the inset shows a schematic of a nanowire in orange and teal, related to the colors of the axes in the graph. Panel b is a magnified version of panel a showing the upper part of the *y*-axis, i.e., nanoparticle In/III ratios of >0.83. This is to highlight the region where a high In/III ratio in the nanoparticle has been reached, allowing a substantial incorporation of In into the nanowire.

The experimental data in Figure 2a show a positive trend, where increasing In content in the nanoparticle (higher values on the *y*-axis) gives an increased In content in the nanowire (higher values on the *x*-axis). For low In content in the nanowire, there is a high positive slope in the data, meaning that there is a large increase in the level of In in the nanoparticle but only a small increase in the level of In in the nanowire. Following this large increase in nanoparticle In/III ratio, the rest of the experimental data show an almost horizontal behavior. In this region, the nanowire In/III ratio covers almost the entire compositional range, while there is only a slight increase in the nanoparticle In/III ratio. This behavior is seen much more clearly in Figure 2b, where the upper part of Figure 2a (nanoparticle In/III ratios of >0.83) is shown.

The high positive slope for low In/III ratios in the nanowire, shown in Figure 2a, indicates that In needs to accumulate in the nanoparticle before it will be incorporated into the growing nanowire. The nanowire In/III ratios acquired in this region are <0.2, meaning that if the nanoparticle is kept in this “buildup” stage, the composition of the nanowire will not reach beyond In_0.2_Ga_0.8_As. The slope levels out at an In/III ratio in the nanoparticle that is >0.9, meaning that >90% of the group III material in the nanoparticle is In, which allows the nanowire to attain In/III ratios of >0.2. In Figure 2a, the region that follows appears to be horizontal, but the expanded scale of Figure 2b reveals that the experimental data show a positive trend across nanowire In/III ratios of >0.2, as well. As stated above, the small positive slope indicates that the difference in nanoparticle In/III ratio across this region is small, while the nanowire covers almost the entire compositional range. This could imply that compositional control is difficult in this region, and small fluctuations in the nanoparticle composition can affect the nanowire composition greatly. At very high In/III ratios in the nanowire, there is again a higher positive slope as the nanoparticle loses all Ga and pure InAs growth is reached.

The experimental data presented in Figure 2 show a relationship between the nanoparticle and nanowire compositions that is similar to what has been predicted in thermodynamically limited growth models.^17,18^ The main difference is that these models predict a miscibility gap, spanning most of the compositions in the “horizontal” region in Figure 2, which would hinder the homogeneous growth of these compositions. In these models, the miscibility gap arises for solid InGaAs below a temperature of 543 °C, which means that it would be present at our much lower growth temperature of 380 °C. Evidently, we have achieved nanowire compositions that span the entire compositional range, strongly indicating that any solid composition can be achieved for In_*x*_Ga_1–*x*_As nanowires. Several kinetically limited growth models have instead shown a suppression of the miscibility gap, allowing the formation of all solid compositions (even if the curve shape often is different from what we have observed here).^14,15^ For this reason, we chose to base our theoretical investigation on a kinetically limited growth model.

Full details of the model are given in the Supporting Information (SI 1: Details about the model); here we present a summary of the important features of the model and compare them to experimental data. We base our model on another very recently published model,^22^ which is kinetically limited at its core and in which the composition of the growing nanowire is determined by the relative rates of attachment of InAs and GaAs to a supercritical nucleus of a certain size. The model also, however, considers thermodynamics to determine, for example, the initial nucleus composition. The shape of the curve that this model produces resembles very much the shape we have seen experimentally, but the miscibility gap is still present. Here we also incorporate surface energies into our revised model and in doing so demonstrate that the miscibility gap is suppressed, producing a full curve that fits the experimental data and is presented by a solid blue line in panels a and b of Figure 2. Surface energies are often omitted in the construction of these types of models, because their contribution is small, but the finite size of the system (the growth interface) motivates their incorporation.

According to our model, the equation that describes the compositional relationship between the nanowire and the nanoparticle is as follows:
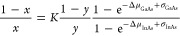
where *x* is the In/III ratio in the nanowire, *K* is a ratio between the attachment coefficients of GaAs and InAs pairs in the nucleus, *y* is the In/III ratio in the nanoparticle, *Δμ*_GaAs_ and *Δμ*_InAs_ are the chemical potential differences related to the addition of GaAs and InAs atomic pairs, respectively, and σ_GaAs_ and σ_InAs_ are composition-dependent surface energy terms related to GaAs and InAs, respectively. A detailed description of how this expression was derived is given in the Supporting Information (SI 1: Details about the model).

As one can see in panels a and b of Figure 2, the model and the experimental data follow each other very well, throughout the entire compositional range. The area that deviates most from earlier models^17,18^ is in the “horizontal” region of the curve, roughly between In/III ratios in the nanowire of 0.2 and 0.9, because this is where the miscibility gap appears. Two main additions have been made to reproduce the experimentally observed behavior. First, the addition of the surface energy terms, which vary linearly with the nanowire In/III ratio, seems to suppress the formation of a miscibility gap. As stated above, the surface energy term is typically omitted in other growth models, an approximation often justified by the relatively large size of the growing layer.^15^ We argue that because the growing layer is finite and limited by the nanowire radius, the surface energy terms give a significant contribution to the composition.

The other addition was an As growth concentration in the nanoparticle, *c*_As_, that increases with an increasing In/III ratio in the nanoparticle, instead of making the simpler assumption that it is constant. When these types of models are constructed, the growth concentration of As is often assumed to be constant and very small, because the solubility is very small in the liquid alloys discussed here. There is, however, a difference between the solubility of As in a Au–Ga alloy and a Au–In alloy, where more As is allowed to be dissolved in the latter case. This increase in solubility with an increasing In/III ratio in the nanoparticle is illustrated in Figure SI 1. The As concentration will affect the chemical potentials, which will induce a slight positive slope across the “horizontal” region, making it more similar to the experimental data as seen in Figure 2b. The surface energy terms also contribute to the slope that arises in the model. In combination, these two additions increase the correlation between the model and the experimental data, and we believe that they are important factors to consider when predicting nanowire growth behavior.

One of the interesting points of the results concerns the high slope of the curve, seen for low In/III ratios in the nanowire in Figure 2a. The behavior observed here indicates that a significant buildup of In to >90% of the total group III in the nanoparticle is necessary before any significant incorporation of In into the nanowire occurs. When such a high In/III ratio in the nanoparticle is reached, the curve flattens, allowing more In to be incorporated into the nanowire. Although the time-dependent compositional evolution has not been directly studied in this paper, the buildup of In in the nanoparticle is expected to take time and could potentially be a limiting step in the compositional control in the growth of ternary nanowires. A great interest in nanowire growth is the ability to grow heterostructures, i.e., homogeneous segments of different materials on top of each other, in the axial direction of the nanowire. To obtain clean interfaces between the segments, the compositional switching in the nanowire needs to be very sharp, which means that this buildup behavior in the nanoparticle must be understood and corrected for.

According to our model, the surface energy terms that vary linearly with nanowire In/III ratio allow the formation of compositions within the miscibility gap. This means that even if the absolute energy difference that these factors contribute is small, their compositional dependency has a significant impact on the achievable compositions. Because it is reasonable to assume that the surface energies would change with composition, we argue that their addition is valid, and that they should be considered when modeling nanowire growth.

Across the “horizontal” region shown in Figure 2b, the difference in In/III ratio in the nanoparticle is very small, which could potentially mean that compositional control is challenging. A slight increase or decrease in the In/III ratio in the particle could result in a large change in the In/III ratio in the resulting nanowire, which could make selection of a specific desired composition difficult. However, the fact that there is a difference in particle In/III ratios in this region is extremely important. If the “horizontal” region had truly been horizontal as predicted in previous models, there would have been no hope of being able to control the composition in the nanowire, because the same In/III ratio in the nanoparticle would give rise to many different nanowire compositions. According to our model, the surface energy terms, as well as the increased growth concentration of As with an increasing In/III ratio in the nanoparticle, are what gives rise to the slight positive slope.

In conclusion, Au-seeded InGaAs nanowires have been studied using *in situ* XEDS, during growth, to measure and understand the correlation between the momentary nanoparticle composition and the composition of the nanowire segment it is growing. According to our experimental investigation, the nanoparticle needs to achieve a high In to total group III ratio for the nanowire to incorporate any significant In. We have found that the occurrence of a predicted miscibility gap in the solid is suppressed, meaning that nanowire compositions across the entire compositional range can be achieved when a large amount of In in the nanoparticle is reached. Our constructed kinetics-based model suggests that the suppression of the miscibility gap occurs due to the change in surface energies that occurs as the solid nucleus of the nanowire is growing. We also observe a slight positive slope across the former miscibility gap region, which our model explains via the addition of the surface energies, as well as an increasing As concentration with an increasing In to total group III ratio in the nanoparticle, which affects the chemical potentials. This slope across the formerly predicted miscibility gap region indicates that deterministic compositional control is possible, because a unique nanoparticle composition is associated with each unique nanowire composition. These findings indicate that the rational design of ternary nanowire materials throughout the entire compositional range is possible, opening the door to larger-scale synthesis of these materials with specific properties designed according to specific application needs.

## Experimental Methods

The experiments were carried out in a Hitachi HF3300S environmental TEM, integrated with a custom-built MOVPE system. The microscope is equipped with an Oxford Instruments SDD X-Max^N^ 80T XEDS system, and the spectra were recorded with sampling times of 60–120 s. Images and movies were recorded using a Gatan OneView IS camera.

Au aerosol particles,^23^ with an average diameter of 30 nm, were deposited with a density of 1 μm^–1^ on MEMS chips made by Norcada Inc. The MEMS chips were mounted in a custom-built sample holder (Hitachi Inc.) equipped with gas injectors, where the gas molecules from the MOVPE system are introduced into the system. To eliminate vibration transfer, the holder is connected to the stainless steel tubing of the MOVPE system via PEEKSiL tubes (polymer-coated quartz capillaries). The MOVPE system uses H_2_ as a carrier gas to transport the metal organic precursors [trimethylgallium (TMGa) and trimethylindium (TMIn) in this case] and has separate channels for the group III metal organics and group V hydrides (AsH_3_ in this case). The gases do not meet until they are at the chip. Blaze software by Hitachi is used to resistively heat the central area of the chips during the experiments. This area consists of a thin SiN_*x*_ film, with circular openings in the material where the electron beam can pass through unobstructed. Growth was initiated from the nanoparticles that had been collected on the film, by heating the sample to 380 °C and supplying the precursors TMIn, TMGa, and AsH_3_ with a gas phase V/III ratio of 1000 and a TMIn to total group III ratio of 0.04. After the nanowires had started growing steadily, the gas flows were changed to achieve desired growth conditions, while keeping the temperature at 380 °C. The subsequently grown nanowires were analyzed as they grew out into the openings in the SiN_*x*_ film. Because there is no substrate with which the direct electron beam can interact in the openings, most of the collected signal comes from the interaction between the electron beam and the nanowire or nanoparticle. This ensures the best spatial resolution when recording images and videos and the least stray X-ray signal from the surrounding film when recording spectra. Further details about the microscope and setup can be found in refs (20) and (24).

## Supporting Information Available

Further details about the model, details about EDX data acquisition and treatment, and an error estimation of the experimental data shown in panels a and b of Figure 2 (PDF)
