# Supplementary material for: Compositional Correlation between the Nanoparticle and the Growing Au-Assisted InxGa1–xAs Nanowire
Source: J Phys Chem Lett. 2021 Aug 4;12(31):7590–5. doi: 10.1021/acs.jpclett.1c02121 (PMC8397339; doi:10.1021/acs.jpclett.1c02121)
Supplement: Supplementary file 1 — jz1c02121_si_001.pdf [file jz1c02121_si_001.pdf]

Supplementary Information for:
Compositional Correlation between the Nanoparticle and the Growing Au-Assisted $In_xGa_{1-x}As$ Nanowire

Robin Sjökvist*[1,2], Daniel Jacobsson[1,2,3], Marcus Tornberg[1,2], Reine Wallenberg[1,2,3], Egor D. Leshchenko[2,4], Jonas Johansson[2,4], Kimberly A. Dick[1,2]

[1]*Centre for Analysis and Synthesis, Lund University, Box 124, 22100, Lund, Sweden.*

[2]*NanoLund, Lund University, Box 118, 22100, Lund, Sweden.*

[3]*National Centre for High Resolution Electron Microscopy, Lund University, Box 124, 22100, Lund, Sweden.*

[4]*Solid State Physics, Lund University, Box 118, 22100, Lund, Sweden.*

*E-mail: robin.sjokvist@chem.lu.se

SI 1: Details about the model

The model is based on the assumption that growth of Au catalyzed $In_xGa_{1-x}As$ nanowires occurs as a result of the incorporation of InAs and GaAs pairs into the growing atomic layer, where the incorporation rates are described by

$$\frac{di}{dt} = W_{InAs}\left(1 - e^{\partial F/\partial i}\right), \qquad \frac{dj}{dt} = W_{GaAs}\left(1 - e^{\partial F/\partial j}\right).$$

Here *i* and *j* are the number of InAs and GaAs pairs in the layer, respectively,[1,2] while *F* is the nucleus formation energy (as discussed later). W_{InAs} and W_{GaAs} are the attachment rates of the pairs, and are assumed to be proportional to the concentration of the attaching species in the droplet, namely c_{In}, c_{Ga} and c_{As}.[3] This gives the attachment rates as

$$W_{InAs} = K_{InAs} c_{In} c_{As}, \qquad W_{GaAs} = K_{GaAs} c_{Ga} c_{As},$$

with attachment coefficients K_{InAs} and K_{GaAs}. The nanowire In/III ratio at a certain time, here denoted by *x*, is given by

$$x = \frac{i}{i+j},$$

since *i* and *j* are directly corresponding to the concentration of In and Ga in the layer. If we assume a steady state composition, dx/dt = 0, the nanowire In/III is given by

$$x = \frac{\frac{di}{dt}}{\frac{di}{dt} + \frac{dj}{dt}},$$

which in effect means that the steady state composition is determined by the incorporation rates of the different pairs. Since the In/III ratio in the nanoparticle, here denoted by y, is defined as

$$y = \frac{c_{In}}{c_{In} + c_{Ga}}$$

we can now write a relation between the nanowire and nanoparticle In/III ratios, based on the previous equations:

$$\frac{1-x}{x} = K\frac{1-y}{y}\frac{\left(1-e^{\frac{\partial F}{\partial j}}\right)}{\left(1-e^{\frac{\partial F}{\partial i}}\right)}.$$

Here, *K* is the ratio between the attachment coefficients:

$$K = \frac{K_{GaAs}}{K_{InAs}}.$$

Now, we turn to the exponents. The nucleus formation energy, F, can be split into two terms:

$$F = -\Delta\mu s + a\sqrt{s}.$$

Here, $\Delta\mu$ is the chemical potential difference between the liquid and solid phase at a particular composition, and its calculation (along with values for the thermodynamic interaction parameters, surface energies, and Gibbs free energies of pure elements and binary compounds) for Au catalyzed nanowire growth can be found elsewhere.[4,5] The nucleus size, *s*, is defined as the number of pairs in the nucleus:

$$s = i + j,$$

and the final variable, *a*, is a surface energy parameter, which is assumed to be a linear combination between the surface energy parameters of the binaries InAs and GaAs based on composition:

$$a = xa_{InAs} + (1-x)a_{GaAs}.$$

The equations for the surface energy parameters are as follows:[6]

$$a_{InAs} = 2\cdot 3^{3/4}\Gamma_{InAs}\sqrt{\Omega^s_{InAs}h_{InAs}}, \qquad a_{GaAs} = 2\cdot 3^{3/4}\Gamma_{GaAs}\sqrt{\Omega^s_{GaAs}h_{GaAs}}.$$

The *Γs* are effective surface energies, which are weighted sums of vapor-solid, liquid-solid and vapor-liquid surface energies, the *Ω* are the volumes of the solid pairs of atoms and the *h* are the nucleus thicknesses. The values used here, along with the calculation of the *Γs*, are found elsewhere.[6]

The partial derivatives of *F* in the exponents are given by:

$$\frac{\partial F}{\partial i} = -\Delta\mu_{InAs} + \sigma_{InAs},$$

$$\frac{\partial F}{\partial j} = -\Delta\mu_{GaAs} + \sigma_{GaAs},$$

where $\Delta\mu_{InAs}$ and $\Delta\mu_{GaAs}$ are the net changes of chemical potential through the addition of an InAs or GaAs pair, respectively, and σ_{InAs} and σ_{GaAs} are surface energy terms given by:

$$\sigma_{InAs} = \frac{1}{\sqrt{s}}\left(a_{InAs}\left(1-\frac{x}{2}\right) - a_{GaAs}\frac{1-x}{2}\right), \qquad \sigma_{GaAs} = \frac{1}{\sqrt{s}}\left(a_{GaAs}\left(1-\frac{1-x}{2}\right) - a_{InAs}\frac{x}{2}\right),$$

where s here denotes an effective average size of the growing layer. This results in a relationship between the composition in nanoparticle and nanowire as

$$\frac{1-x}{x} = K\frac{1-y}{y}\frac{\left(1-e^{-\Delta\mu_{GaAs}+\sigma_{GaAs}}\right)}{\left(1-e^{-\Delta\mu_{InAs}+\sigma_{InAs}}\right)}.$$

The addition of the surface energy terms leads to the suppression of the miscibility gap, which gives a much better fit to the experimentally observed data. The miscibility gap would otherwise span most of the "horizontal" region seen in Figure 2 (a) in the main text, and suggest that these compositions could not be grown as a homogeneous solid.

When applying this model, some parameters are used as fitting parameters. The attachment coefficient ratio, K, is unknown, as well is the surface energy parameters (since the effective surface energies Γ_{InAs} and Γ_{GaAs} are unknown), a_{InAs} and a_{GaAs}, the average nucleus size, s, and the As concentration in the particle, c_{As}. The As concentration will affect the chemical potentials, $\Delta\mu$. For the calculations, the values K = 3 and $c_{As} = 10^{-4}\exp(2.8\cdot y^2)$ were used. The surface energies are weak linear functions of the liquid composition so that $a_{InAs}/(\sqrt{s}RT)$ increases linearly from 3.53 to 3.66 and $a_{GaAs}/(\sqrt{s}RT)$ from 4.05 to 4.16 as y increases from 0 to 1. Even though the exact value of the As concentration has to be estimated, the assumption that the growth concentration is increasing with increasing In/III ratio in the nanoparticle is reasonable, since the solubility of As in the nanoparticle is increasing with increasing In/III ratio. This is illustrated in Figure SI 1. The effect of this assumption, as stated in the main text, is that the "horizontal" region, as seen in Figure 2 (a) and (b) in the main text, gets a slight positive slope. This is what is observed experimentally.

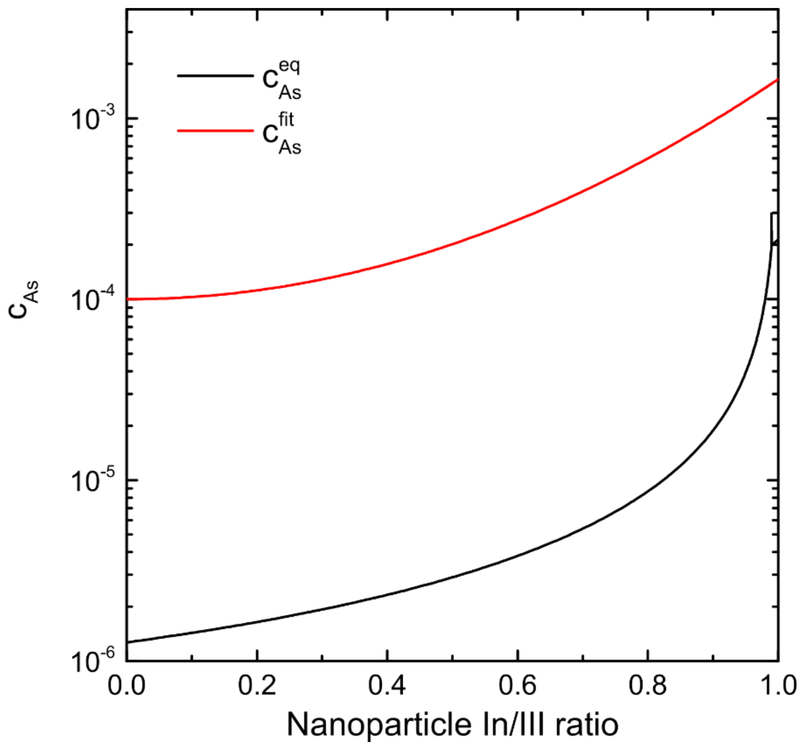

Figure SI 1: The As concentration dependence on the nanoparticle In/III ratio, y. The black curve shows how the solubility changes, while the red curve shows the equation used in the model.

SI 2: The experimental data

Table SI 1, presented in section SI 5: Tables, shows a summary of the compositions in nanowire and nanoparticle, measured using XEDS. The values are from the AZtec software, after normalization to As + Ga + In + Au = 100% and after conversion from wt% to at%.

For the nanoparticle measurements, the arsenic percentage was used as an indication for the level of stray signal that was recorded from the wire during the measurement. This was because the arsenic is expected to have very low solubility in the nanoparticle, and ideally should not show up in the XEDS measurements of the nanoparticle. The level of arsenic is generally low, with some exceptions showing 4 at% and above. The As signal also showed a slight increase throughout experiment days, which is thought to be a result of stray signal from the surrounding surface layer growth that gets increasingly worse as the experiment progresses. During the recording of the nanowire composition, the Au signal was used as an indication that the beam had been placed a sufficient distance from the nanoparticle. Generally, the measured gold signal was low.

As can be seen in Table SI 1, the arsenic signal measured in the nanowire was not always 50 at%, which is the expected, stoichiometric, value. Similarly, the measured In and Ga should equal to 50 at% as well. The error can in part arise from the small gold signal measured, skewing the percentages somewhat, but what seems to be of greater importance is the channeling effect. This effect arises if the sample is tilted close, but not perfectly, to a zone axis. Since InGaAs is a polar compound, this can lead to preferential x-ray emission, where either a group III or group V layer in the crystal is favored and therefore exaggerated.[7] We expect this effect to have little influence on the presented results in the main text, since the two group III elements are occupying the same positions in the nanowire, and there should

therefore not be any preference in their excitation related to channeling. The reported In/(In+Ga) ratio is therefore assumed to be correct.

SI 3: XEDS error estimation

A measurement error estimation was performed for the XEDS data. The XEDS-software, AZtec (Oxford Instruments), provides a measurement error for individual elements. This error, however, is given as an error on the Wt% measured of each element. To transform this error to a final error for the In/(In+Ga) ratio, the following procedure was used:

From AZtec, for each measurement, the Wt%, at% and err(Wt%) was extracted for each element, In, As, Ga and Au. The relative size of the error, r, was calculated for each individual element

$$r = \frac{err(Wt\%)}{Wt\%}.$$

This enabled us to calculate the error expressed instead in atomic percent

$$err(at\%) = r \cdot at\%.$$

The error here represents an upper and lower limit of what could be the true value for each element in a measurement. Therefore, in order to get the error for the ratio between In and total group III, In/(In+Ga), a matrix was constructed, where the measured value of Ga and In was stepped incrementally between their respective lowest and highest values, given by the error value. From the matrix, the lowest and highest value of the ratio In/(In+Ga) was selected to represent the lowest and highest bounds of the error bar, respectively. In Table SI 2, the values of In/(In+Ga) used in the construction of Figure 2 in the main text are shown, along with the lower and upper bounds of the error bars. This is also presented as a graph in Figure SI 2.

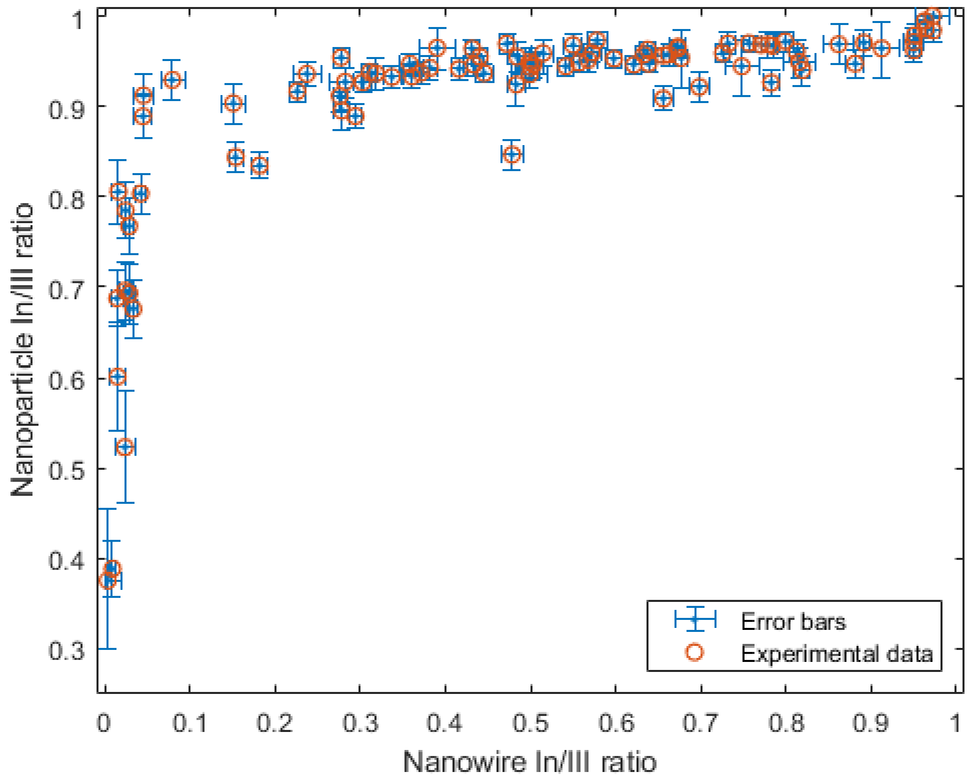

Figure SI 2: The experimental data presented in Figure 2 (a) and (b) in the main text, along with the calculated upper and lower errors bounds from Table SI 2, presented as error bars.

SI 4: Distorted beam for XEDS

While measuring the composition in the nanowire, we realized that the region from which the x-ray signal was recorded could be minimized by distorting the shape of the beam to a disc, by introducing condenser lens astigmatism. The disc would be placed along the flat nanowire-particle interface, to minimize the spread of the beam in the axial direction and therefore limit the thickness of the nanowire segment illuminated by the beam. It would also mean that an XEDS measurement could be conducted of a segment closer to the interface, without illuminating the nanoparticle. The introduced astigmatism was corrected for after the measurement, so as to not affect the imaging. Figure SI 3 illustrates the difference between the round and the flat beam.

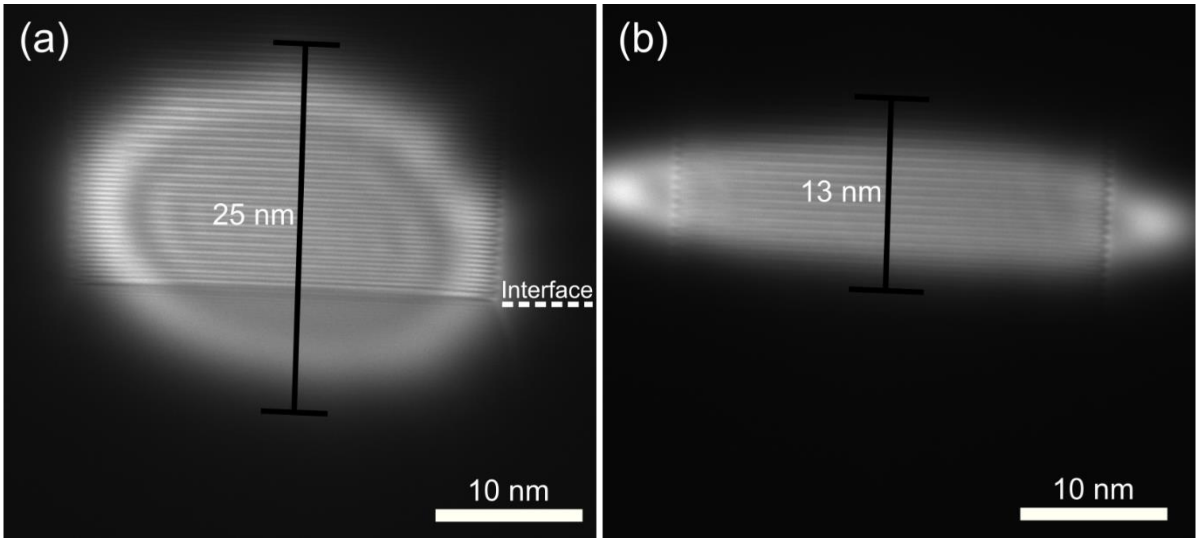

Figure SI 3: Images recorded of a nanowire using a round (a) or a flat (b) beam. In (a) the distance illuminated in the axial direction of the nanowire is approximately 25 nm, and the nanoparticle/nanowire interface is indicated. In (b) the spread of the beam is limited, meaning that only roughly 13 nm are illuminated in the axial direction. The beam is not hitting the nanoparticle in this case, which means that an XEDS measurement of the nanowire can be conducted.

SI 5: Tables

Table SI 1: A summary of the compositional data acquired through XEDS, used when calculating the experimental data, presented in the main text.

	**Nanoparticle composition [at%]**				**Nanowire composition [at%]**			
Measurement number	**Ga**	**As**	**In**	**Au**	**Ga**	**As**	**In**	**Au**
1	13.41	0.11	14.71	71.78	47.67	50.2	1.14	0.99
2	16.79	1.69	10.1	71.42	47.47	49.95	0.15	2.42
3	15.98	1.72	10.16	72.14	50.00	48.25	0.44	1.31
4	12.89	1.22	19.44	66.46	50.68	47.74	0.76	0.82
5	9.77	1.1	21.49	67.65	49.71	47.91	0.72	1.65
6	9.62	1.97	22.01	66.39	48.71	48.09	1.18	2.02
7	7.39	2.07	24.37	66.18	49.75	47.38	1.45	1.42
8	9.68	1.15	21.83	67.34	50.37	46.49	1.48	1.66
9	6.98	2.28	25.42	65.33	51.08	46.03	1.27	1.62
10	6.68	1.26	27.68	64.38	49.81	47.43	0.8	1.97
11	6.94	1.72	28.37	62.97	48.86	47.1	2.14	1.9
12	4.19	0.97	33.54	61.3	49.06	47.18	2.29	1.47
13	3.41	1.44	35.39	59.76	46.09	49.79	2.18	1.94
14	2.36	0.24	39.99	57.41	11.8	50.76	35.1	2.34
15	0.24	0.99	45.32	53.45	2.12	39.26	55.88	2.74
16	0.63	1.9	44.23	53.24	2.00	40.48	54.39	3.13
17	1.29	0.89	43.41	54.41	2.66	44.04	50.77	2.54

18	0.72	1.01	44.19	54.08	1.38	45.84	49.74	3.04
19	1.78	1.45	45.29	51.48	2.63	44.47	50.65	2.25
20	0.00	1.32	44.99	53.69	1.66	36.27	58.58	3.5
21	0.92	1.77	42.02	55.29	2.67	42.32	52.42	2.58
22	1.54	1.95	41.39	55.12	4.32	48.27	45.26	2.16
23	1.36	2.55	42.2	53.89	7.64	41.88	47.73	2.75
24	1.34	3.11	43.77	51.78	7.41	28.62	61.18	2.78
25	2.34	3.77	42.12	51.77	4.4	60.31	32.73	2.55
26	2.3	1.38	37.72	58.59	31.1	47.19	19.23	2.49
27	1.53	2.3	41.91	54.26	30.00	48.15	19.2	2.65
28	2.47	2.08	36.39	59.05	38.66	41.09	18.06	2.19
29	3.89	3.19	36.4	56.51	42.49	48.15	7.54	1.83
30	3.14	3.29	34.61	58.95	38.06	48.51	11.11	2.32
31	2.71	2.84	39.61	54.83	37.22	49.94	11.57	1.27
32	3.35	2.39	42.62	51.64	35.2	48.95	13.84	2.01
33	1.99	3.19	42.21	52.61	27.73	48.05	21.83	2.39
34	2.92	2.08	42.36	52.64	25.7	46.5	25.64	2.16
35	9.55	2.13	19.9	68.42	47.57	49.32	1.63	1.49
36	5.29	1.86	29.17	63.68	25.99	47.95	23.84	2.23
37	5.19	1.58	28.03	65.2	41.1	49.67	7.46	1.77
38	2.48	1.86	32.55	63.11	43.6	51.66	3.75	0.98
39	2.17	2.45	38.82	56.57	31.17	48.98	17.4	2.44
40	2.23	3.56	35.06	59.15	8.04	50.93	36.52	4.5
41	2.8	2.89	35.09	59.22	10.35	47.73	37.29	4.63
42	3.41	3.79	29.14	63.67	34.19	48.83	13.17	3.82
43	1.99	2.76	37.08	58.17	7.98	51.79	35.34	4.89
44	1.82	4.01	38.4	55.77	26.25	46.86	24.66	2.23
45	1.96	2.27	40.82	54.94	37.4	46.33	14.36	1.91
46	2.9	3.67	40.38	53.04	32.79	46.47	18.52	2.22
47	2.41	4.02	39.95	53.61	25.36	46.45	25.38	2.81
48	2.12	3.52	41.5	52.86	21.92	46.51	28.78	2.8
49	1.74	3.6	40.83	53.83	21.83	46.46	29.22	2.5
50	2.2	4.35	40.52	52.93	22.19	47.81	27.9	2.1
51	5.67	1.06	28.59	64.69	37.39	52.43	8.3	1.88
52	3.94	1.26	31.63	63.17	41.1	40.26	17.14	1.5
53	1.29	0.87	40.77	57.07	27.89	45.55	24.93	1.63
54	2.39	1.84	40.52	55.25	23.17	47.46	27.39	1.97
55	1.63	1.13	42.1	55.14	18.26	47.31	32.17	2.25
56	1.61	1.59	43.24	53.56	12.21	60.77	25.17	1.86
57	1.35	2.27	42.95	53.43	13.02	49.06	35.67	2.26
58	1.35	1.71	40.98	55.96	11.07	49.45	37.25	2.23

59	1.31	2.44	42.2	54.05	12.8	44.67	39.82	2.71
60	1.65	3.3	40.62	54.42	9.35	47.54	40.54	2.57
61	1.39	3.08	42.6	52.93	11.33	45.95	39.98	2.74
62	1.2	3.28	41.73	53.78	10.21	46.85	40.68	2.25
63	3.54	3.57	36.47	56.42	40.87	41.69	15.57	1.86
64	1.79	3.56	41.76	52.89	14.03	47.26	37.16	1.55
65	1.47	3.55	41.79	53.19	16.93	46.45	34.88	1.74
66	2.26	4.02	40.55	53.16	18.84	46.02	33.32	1.82
67	1.79	4.51	40.12	53.58	18.69	47.26	32.26	1.78
68	2.01	4.93	40.5	52.56	20.48	47.12	30.49	1.91
69	2.27	4.42	39.78	53.53	19.49	46.62	32.00	1.89
70	2.09	3.05	42.85	52.01	16.24	47.49	34.06	2.21
71	1.86	2.73	41.81	53.61	17.34	46.37	33.99	2.3
72	1.4	2.21	41.04	55.35	11.16	46.14	40.64	2.06
73	3.1	4.12	37.77	55.01	27.21	44.57	25.47	2.75
74	1.48	2.03	44.6	51.89	23.42	44.67	28.62	3.29
75	1.74	2.51	40.61	55.13	24.72	46.15	26.23	2.9
76	2.36	2.52	41.55	53.58	25.09	46.88	25.44	2.59
77	2.37	2.54	41.59	53.5	29.3	45.52	22.41	2.77
78	2.17	3.27	40.2	54.36	25.61	46.61	25.46	2.33
79	2.85	1.96	33.42	61.77	15.06	46.47	34.88	3.59
80	3.59	2.32	35.65	58.44	16.67	47.88	31.81	3.63
81	1.97	2.24	43.16	52.63	17.00	47.69	32.46	2.85
82	1.19	1.97	42.71	54.12	20.67	48.49	28.3	2.54
83	1.53	1.88	40.82	55.77	28.37	47.44	21.54	2.65
84	2.68	2.38	39.68	55.26	29.74	43.26	23.9	3.09
85	2.4	2.9	38.67	56.03	27.79	49.82	19.79	2.6
86	2.56	3.27	38.13	56.03	32.54	45.59	19.2	2.67
87	2.53	2.68	38.18	56.61	32.5	49.97	14.7	2.83
88	2.93	2.29	37.04	57.74	35.48	46.62	15.4	2.5
89	2.74	2.64	37.88	56.75	33.22	47.1	16.9	2.78

Table SI 2: The calculated In/(In+Ga) ratios that was used for Figure 2 (a) and (b) in the main text, along with the calculated lower and upper bounds for the error bars used in the construction of Figure SI 2.

	**Nanoparticle ratio**			**Nanowire ratio**		
Measurement number	**In/(In+Ga)**	**Lower bound**	**Upper bound**	**In/(In+Ga)**	**Lower bound**	**Upper bound**
1	0.523	0.461	0.585	0.023	0.012	0.035
2	0.376	0.300	0.454	0.003	0.000	0.019
3	0.389	0.358	0.419	0.009	0.004	0.013
4	0.601	0.541	0.661	0.015	0.005	0.025

5	0.687	0.656	0.718	0.014	0.008	0.021
6	0.696	0.663	0.728	0.024	0.016	0.031
7	0.767	0.736	0.798	0.028	0.023	0.034
8	0.693	0.659	0.726	0.029	0.022	0.035
9	0.785	0.753	0.815	0.024	0.019	0.030
10	0.806	0.769	0.841	0.016	0.008	0.024
11	0.803	0.781	0.825	0.042	0.036	0.048
12	0.889	0.864	0.913	0.045	0.034	0.055
13	0.912	0.888	0.935	0.045	0.034	0.057
14	0.944	0.912	0.974	0.748	0.728	0.769
15	0.995	0.977	1.000	0.963	0.952	0.975
16	0.986	0.975	0.997	0.965	0.958	0.971
17	0.971	0.956	0.986	0.95	0.939	0.961
18	0.984	0.970	0.997	0.973	0.966	0.980
19	0.962	0.950	0.974	0.951	0.945	0.957
20	1.000	1.000	1.000	0.972	0.952	0.992
21	0.979	0.965	0.992	0.952	0.944	0.959
22	0.964	0.932	0.994	0.913	0.892	0.933
23	0.969	0.946	0.990	0.862	0.844	0.880
24	0.97	0.957	0.983	0.892	0.886	0.898
25	0.947	0.931	0.963	0.881	0.870	0.892
26	0.943	0.930	0.955	0.382	0.374	0.390
27	0.965	0.941	0.988	0.39	0.369	0.412
28	0.936	0.919	0.954	0.318	0.309	0.328
29	0.903	0.880	0.926	0.151	0.137	0.164
30	0.917	0.906	0.928	0.226	0.216	0.236
31	0.936	0.922	0.950	0.237	0.221	0.254
32	0.927	0.910	0.943	0.282	0.263	0.302
33	0.955	0.944	0.965	0.44	0.431	0.450
34	0.936	0.921	0.950	0.499	0.478	0.521
35	0.676	0.643	0.708	0.033	0.027	0.039
36	0.846	0.829	0.864	0.478	0.465	0.491
37	0.844	0.827	0.861	0.154	0.145	0.162
38	0.929	0.907	0.950	0.079	0.065	0.094
39	0.947	0.935	0.959	0.358	0.347	0.370
40	0.94	0.923	0.957	0.82	0.810	0.829
41	0.926	0.912	0.940	0.783	0.773	0.792
42	0.895	0.875	0.915	0.278	0.268	0.288
43	0.949	0.932	0.965	0.816	0.797	0.834
44	0.955	0.938	0.971	0.484	0.474	0.495
45	0.954	0.943	0.965	0.277	0.268	0.287

46	0.933	0.921	0.945	0.361	0.351	0.371
47	0.943	0.929	0.956	0.5	0.490	0.511
48	0.951	0.938	0.965	0.568	0.557	0.578
49	0.959	0.949	0.969	0.572	0.562	0.582
50	0.949	0.938	0.959	0.557	0.546	0.568
51	0.835	0.820	0.849	0.182	0.172	0.191
52	0.889	0.876	0.903	0.294	0.288	0.300
53	0.969	0.959	0.980	0.472	0.464	0.480
54	0.944	0.934	0.955	0.542	0.533	0.551
55	0.963	0.953	0.972	0.638	0.629	0.647
56	0.964	0.955	0.973	0.673	0.664	0.683
57	0.97	0.958	0.981	0.733	0.725	0.740
58	0.968	0.959	0.977	0.771	0.764	0.778
59	0.97	0.961	0.979	0.757	0.750	0.764
60	0.961	0.948	0.973	0.813	0.805	0.820
61	0.968	0.954	0.983	0.779	0.771	0.787
62	0.972	0.961	0.983	0.799	0.790	0.808
63	0.912	0.895	0.928	0.276	0.268	0.284
64	0.959	0.949	0.968	0.726	0.718	0.734
65	0.966	0.958	0.974	0.673	0.664	0.682
66	0.947	0.937	0.957	0.639	0.631	0.647
67	0.957	0.948	0.967	0.633	0.624	0.643
68	0.953	0.943	0.962	0.598	0.589	0.608
69	0.946	0.935	0.957	0.621	0.613	0.630
70	0.953	0.921	0.984	0.677	0.670	0.684
71	0.957	0.945	0.970	0.662	0.656	0.668
72	0.967	0.954	0.980	0.785	0.780	0.789
73	0.924	0.901	0.946	0.483	0.473	0.494
74	0.968	0.955	0.980	0.55	0.539	0.561
75	0.959	0.944	0.973	0.515	0.502	0.528
76	0.946	0.932	0.960	0.503	0.493	0.514
77	0.946	0.933	0.959	0.433	0.422	0.445
78	0.949	0.932	0.965	0.499	0.488	0.509
79	0.921	0.904	0.938	0.698	0.687	0.710
80	0.909	0.895	0.922	0.656	0.645	0.667
81	0.956	0.948	0.964	0.656	0.645	0.668
82	0.973	0.964	0.982	0.578	0.566	0.589
83	0.964	0.954	0.974	0.432	0.423	0.440
84	0.937	0.927	0.946	0.446	0.434	0.457
85	0.942	0.929	0.954	0.416	0.406	0.426
86	0.937	0.925	0.949	0.371	0.361	0.381

87	0.938	0.928	0.948	0.311	0.302	0.321
88	0.927	0.915	0.938	0.303	0.293	0.312
89	0.933	0.920	0.944	0.337	0.327	0.347

References

(1) Johansson, J.; Ghasemi, M. Kinetically Limited Composition of Ternary III-V Nanowires. *Phys. Rev. Mater.* **2017**, *1* (4), 040401. https://doi.org/10.1103/PhysRevMaterials.1.040401.

(2) Dubrovskii, V. G. *Nucleation Theory and Growth of Nanostructures*; NanoScience and Technology; Springer Berlin Heidelberg: Berlin, Heidelberg, 2014. https://doi.org/10.1007/978-3-642-39660-1.

(3) Leshchenko, E. D.; Johansson, J. Role of Thermodynamics and Kinetics in the Composition of Ternary III-V Nanowires. *Nanomaterials* **2020**, *10* (12), 1–15. https://doi.org/10.3390/nano10122553.

(4) Leshchenko, E. D.; Ghasemi, M.; Dubrovskii, V. G.; Johansson, J. Nucleation-Limited Composition of Ternary III–V Nanowires Forming from Quaternary Gold Based Liquid Alloys. *CrystEngComm* **2018**, *20* (12), 1649–1655. https://doi.org/10.1039/C7CE02201H.

(5) Johansson, J.; Ghasemi, M. Composition of Gold Alloy Seeded InGaAs Nanowires in the Nucleation Limited Regime. *Cryst. Growth Des.* **2017**, *17* (4), 1630–1635. https://doi.org/10.1021/acs.cgd.6b01653.

(6) Johansson, J.; Leshchenko, E. D. Zinc Blende and Wurtzite Crystal Structure Formation in Gold Catalyzed InGaAs Nanowires. *J. Cryst. Growth* **2019**, *509* (January), 118–123. https://doi.org/10.1016/j.jcrysgro.2019.01.002.

(7) Ek, M.; Lehmann, S.; Wallenberg, R. Electron Channelling: Challenges and Opportunities for Compositional Analysis of Nanowires by TEM. *Nanotechnology* **2020**, *31* (36), 364005. https://doi.org/10.1088/1361-6528/ab9679.
